# Round the Hospitals

**Published:** 1919-12-27

**Authors:** 


### ROUND THE HOSPITALS.

The King held an Investiture on December 17 in the Ballroom of Buckingham Palace and personally conferred decorations on members of the nursing services as follows:—Bar to the Royal Red Cross: Miss Sarah Brown, Q.A.I.M.N.S. (R.). Royal Red Cross and Bar: Miss Una Lee, Q.A.I.M.N.S. (R.). Royal Red Cross, First Class: Miss Lavina Badger. Miss Ina Humfrey, Miss Louise Remnant, and Miss Kate Underwood, Q.A.I.M.N.S. (R.); Miss Pauline Barnard and Miss Isabel Eveleigh, T.F.N.S. Royal Red Cross, Second Class: Miss Joan Wells. Q.A.I.M.N.S.; Miss Lily Baldwin, Miss Mildred Beale, Miss Elizabeth Betts, Miss Charlotte Black. Miss Beatrice Brice, Miss Eileen Byrne, Miss Kathleen Cambridge, Miss Norah Connolly, Miss Mary Farmer, Miss Helen Fisher, Miss Margaret Griffith, Miss Beatrice Hall, Miss Elizabeth Lee, Miss Agnes Lithgow, Miss Christian Valentine, Miss Sarah Wadsworth, Miss Jessie Warnock-Walker, Miss Mary Welch, and Miss Audrey Wellington,

**ROUND THE HOSPITALS**—(*continued*).

Q.A.I.M.N.S. (R.); Miss Alice Bunch, Miss Effie White, and Miss Nona White, T.F.N.S.; Miss Helen Walters, C.N.S.; Miss Dorothy Sweet and Miss Joan van der Weyer, B.R.C.S.; Miss Kate Bradford and Miss Grace Broadhead, St. John's A.B.; Mrs. Letitia Breese, Miss Margaret Gale, Miss Kathleen Grimbley, Mrs. Harriet Llewellyn, Mrs. Kate Talbot, and Miss Una Ward, V.A.D. Military Medal: Miss Lily Gregory, V.A.D. All these ladies, with Miss A. B. Smith, R.R.C., were subsequently received at Marlborough House by Queen Alexandra.

---

The King held a second Investiture last week on December 19 in the Ballroom of Buckingham Palace, and personally conferred decorations on the following members of the nursing services:—Bar to the Royal Red Cross: Miss Elizabeth Dowse, Q.A.I.M.N.S. (R.). Royal Red Cross and Bar: Miss Katherine Skinner, Q.A.I.M.N.S. Royal Red Cross, First Class: Miss Gertrude Aitchison, Miss Gwendoline Hughes, Miss Margaret Ram, and Miss Cecilia Stevens, Q.A.I.M.N.S. Miss Florence Harley, Q.A.I.M.N.S. (R.); Miss Winifred Attenborough, T.F.N.S.; Miss Maude Inness, V.A.D. Royal Red Cross, Second Class: Miss Agnes A'Hern, Miss Margaret Anderson, Miss Mary Andrew, Miss Edith Aylett, Miss Lucy Brawn, Miss Alice Fletcher, Miss Florence Hyndman, Miss Penelope Roberts, and Miss Annetta Sinclair, Q.A.I.M.N.S. (R.); Miss Portia Batley, Miss Marion Crosbie, Miss Mildred Edwards, Miss Maude Emberson, Miss Florence Henry, and Miss Isabella Stratton, T.F.N.S.; Miss Lilian Branston, B.R.C.S.; Mrs. Olympia Honeyball, Miss Evelyn Sanders, and Miss Mary Weaver, Civil and W.H.; Miss Phyllis Bruce, Mrs. Nellie Dempster, Miss Mary Earp, Miss Elizabeth Henry, Miss Nona Molson, Miss Kate Slater, Mrs. Dorothy Sumner, Miss Dorothea Wood, and Miss Lucy Wood, V.A.D.. Military Medal: Miss Nellie Galvin, Q.A.I.M.N.S. (R.); and Miss Daisy Dobbs, T.F.N.S. All these ladies were subsequently received at Marlborough House by Queen Alexandra.

---

The following members of the nursing services were awarded distinction in the final list of war decorations:—Bar to the Royal Red Cross: Miss S. A. Stevenson, R.R.C., Matron T.F.N.S. (substituted for notification of the R.R.C., Second Class in *Gazette* of October 24, 1917); Miss E. Russell, R.R.C., matron; Miss E. A. Congers, C.B.E., R.R.C., Matron-in-Chief Australian A.N.S. Royal Red Cross, First Class: Miss E. M. Beverley, Matron Epping M.A.H.; Miss E. S. Clark, Matron Whipps Cross W.H.; Miss L. Drewitt, A.R.R.C., Matron Gen. Inf., Macclesfield; Miss E. M. Fox, Matron B.R.C.S.; Miss A. Guest, Matron A.M.H., Birkenhead; Miss R. Paul, Matron Wellington Inf. A.N.; Miss E. Price, Acting-Matron A.R.R.C., Highfield M.H., Liverpool. Canadian Army Medical Corps: Miss A. G. Hogarth, A.R.R.C.; Miss B. A. Merriman, A.R.C.C.; Miss E. W. Odell, A.R.R.C. Australian A.N.S.: Miss A. M. Cooper, A.R.R.C.; Miss L. C. Pratt, A.R.R.C.; Miss E. M. Strickland, A.R.R.C. New Zealand A.N.S.: Miss S. L. Clark, Miss A. C. Ingles, A.R.R.C. The Associateship of the R.R.C. was granted to 26 English Army sisters and nurses, among whom we note several attached to the Whipps Cross Hospital; 22 Canadian Army sisters and nurses, two Australian Army matrons; six New Zealand Army sisters; two Newfoundland sisters, and two sisters from West Africa.

---

The Nurses' Registration Bill sailed through its second reading in the House of Lords with a favourable wind. The only doubtful voice was that of Lord Knutsford, who acclaimed registration as foredoomed to failure and urged the importance of making it compulsory instead of voluntary. In this he exemplified the tendency of all late converts to outdo the orthodox in championship of the cause. We are inclined to think that Lord Knutsford is not far wrong in pointing to previous attempts at voluntary state registration as failures. But it does not follow that British nurses will not succeed where others have failed. In one respect the nursing profession in this country starts registration at a great advantage. The profession is already organised. All the training-schools are in accord. They adopt registration as the sign and seal of union. Instead of having to wait and whip in reluctant training centres, we have them all prepared to give a hearty welcome to the new regulations. The main problem which devolves on the new Nursing Council is to determine the bait, for it comes to this, which shall be offered to women already certificated to register their name, in order that the unity of the profession may be unbroken. The *vis inertiæ* which keeps people from participating in movements on which their own future may depend has to be reckoned with. But there are some wise heads among us, and we must look to them to see that registration is made, not only popular, but indispensable. The three Registration Bills (No. 2, Scotland, and Ireland) have now passed through the House of Lords, whose amendments were considered in the Commons on December 19, and agreed to without alteration, except in the case of the Bill for Ireland, where a further amendment was made.

---

A vast scheme of reorganisation as regards the health services of London is now under consideration by the London County Council. It is recommended that the Council shall become the statutory authority to survey the health services within the county and to supervise and organise (though not necessarily to provide) those for which the local authorities are made responsible. The Council would be the sole central institutional authority for the County of London. It would appropriate, as and when thought fit, institutions transferred from the Poor Law, and take over *en bloc* the institutions of the Metropolitan Asylums Board. The formation of a Central Council of London Hospitals is fore-

**ROUND THE HOSPITALS—(*continued*).**

casted, representing all the voluntary London hospitals, and including representatives of the Ministry of Health and the L.C.C. Such a hospital Council would have a recognised position in the general scheme for health administration in London.

---

It is impossible to exaggerate the importance to the nursing profession of this reorganisation of health services. The difficulties and hardships under which nurses labour are due in very large part to defects of administration. While well-managed hospitals and Poor-Law infirmaries treat the nursing staff liberally, others are miserably indifferent to the hours they work, their housing, recreation, food, and pay. A central body could gradually raise the standard until the profession became transformed and abuses disappeared. Under the M.A.B. an eight-hours' day has been instituted with one day in seven off duty. Can it be believed that the Central Authority would prove less enlightened than the Board which is transferred to its control? Whichever way we look at the new scheme it appears full of promise to nurses.

---

We take the following from the personal columns of the *Times*, December 20:—" Will the Nurse H. who was given signet ring in 1913 by an Indian Princess please communicate with No. 40 Scripps' Agency, South Molton Street, W. 1."

---

Birmingham has taken a great forward step in the institution of a forty-eight hour week, or rather of a ninety-six hour fortnight, in its municipal institutions. It is deeply to be regretted that the Infirmary Committee have reported against the adoption of this reform. Their reason for so doing is the impossibility of providing residential accommodation for the additional nursing and domestic staffs required without the expenditure of a very large capital sum in the erection of buildings. The Committee reports, however, that the average number of working hours for the nursing and domestic staffs can be reduced to fifty-four weekly. The apparent hardship of arranging for nurses to work about one hour a day longer than the other employees of the Board can only be alleviated by taking steps to ensure that some if not all of this time is devoted directly to the advancement of their knowledge of their profession.

---

The Croydon Poor-law Infirmary is now, for the first time since the war began, rejoicing in a full nursing staff. It is an appropriate moment for returning thanks to those who have so bravely weathered the storm, and the Chairman gave utterance at the last meeting of the Board to a formal but very cordial expression of the debt which the Guardians and the town generally owes to the medical superintendent, the matron, Miss Pagen, and her nurses.

A discussion has been taking place in *The Common Cause* on the ethics of registration for nurses and the effect of " the tradition of the convent " on the characters of nurses. Miss Helen G. Klaassen has some pertinent remarks on the subject. We entirely agree with her that hospital governors have something to learn from convents. Though women in a convent take vows of poverty they are looked after in illness and old age. " They do not have to dread old age." We agree also in her concluding remarks: " What nurses want more than anything is to lift up their hearts and find themselves able to take an interest in the management of their profession and in the work of the new Health Ministry."

---

An interesting appeal case has just been settled in the High Court of Justice which illustrates the importance of getting names exactly right, a point in which we fear " the unbusinesslike nurse " is not always free from blame. Mr. Nicholay, who died in 1901, left 10-174ths of his residuary estate to a society, which he described as " The Society for the Prevention of Cruelty to Women and Children." The money is claimed by two societies, " The Society for the Prevention of Cruelty to Children " and " The Associated Societies for the Protection of Women and Children," and after a series of legal proceedings extending over some years, and culminating in the appeal to the High Court, has been finally adjudged to the " Associated Societies." The moral is that persons consulted about the claims of institutions or societies in which they are interested should set down in writing for the information of their beneficent friends the exact style and title, and not imagine that " National," for instance, will do just as well as " Royal," or " Hospital " instead of " Infirmary."

---

It is proposed to establish a hostel for unmarried mothers and deserted wives with their babies in the St. Pancras Borough, in which they may live and pay for their maintenance by the proceeds of daily work. The scheme is devised to enable unprotected mothers to have their infants with them instead of putting them out to nurse. The Borough has four day nurseries, which it is proposed to subsidise from municipal funds. While somewhat doubtful about the expediency of putting unmarried mothers and deserted wives on the same level, we recognise that the practice of pouring cold water on plans of this nature has gone too far. The need is for experiment in a spirit of courage and faith in human nature. Dr. Higgins, the medical officer of health for the district, appears to have got at the root of the matter, and his venture will be watched with great interest by other boroughs.

---

The members of the Ladies' Association attached to the Hampstead General Hospital testified their good will in a very practical manner at their annual meeting. Miss Lilian Braithwaite made an eloquent appeal to the meeting for gifts, and specified the need for eighty blankets at 17s. 6d. each and

**ROUND THE HOSPITALS—(*continued*).**

a dinner wagon. Two ladies at once volunteered to defray the cost of the dinner wagon, and the money for the blankets was promptly made up, which shows how far a persuasive and (in the best sense) artful way of putting things can carry an audience. The Countess de Torby was in the chair, and the Grand Duke Michael of Russia accompanied her.

---

The V.A.D.s have had a good farewell both at Newcastle and at Lincoln. Sir Thomas Oliver presided at the Newcastle meeting, and he paid a well-deserved tribute to the matrons who directed the work and the nurses who worked under them. Miss Tidswell, Mrs. Darley, Mrs. Sinclair Fraser and Miss Hutchinson were marked out for special mention. Nearly 1,000 patients were treated, many difficult and slow-healing cases having been passed on from the Northern General Hospital. Medals were presented to all the workers, and Miss Hutchinson's well-deserved popularity was attested by the gift of a gold signet ring, with shield of white enamel bearing the red cross. At Lincoln medals were presented by Lady Monson to thirteen nurses for service during the war and Lt.-Col. Lambert, Asst.-Brigade Commissioner for Lincoln, eulogised the work of the nurses and commended especially those who had done ambulance work at the Great Northern Station and were often there all night.

---

The annual prize-giving at the Bristol General Hospital was a very cheerful event. Everyone had the feeling of having got through the worst, with brighter times in store. Everyone was happy at the return of the war sisters, among whom are Miss Cox and Miss Sampson, and rejoiced at the resumption of normal activities. Mr. Herbert Baker presided as Chairman of the Committee, and was able to announce that arrangements had been made for giving the nurses more time off duty, and that the Committee were fully alive to the changes which were going on in the nursing profession, and ready to comply with any proposal for betterment put forward by the medical staff. He thanked all the officers, and especially the matron and the assistant-matron. His remark that they had a matron second to none stirred the gathering to enthusiastic applause. More than one speaker gave his testimony to the excellence of the nursing in the institution. The prizes were distributed by Mrs. Michell Clarke, daughter of Dr. Clarke, whose memory is so highly honoured in the hospital. Gold Medal, Nurse Dorothy Dingle; Silver Medal, Nurse Julie Hillier; Nurse Culverwell Prize for the best nurse of the year, Nurse Dorothy Dingle. First Prizes: Surgical Nursing, Nurse Beatrice Worral; Medical Nursing, Nurse Margaret Glasbrook; Anatomy and Physiology, Nurse Elizabeth Macleod; Practical Nursing, Miss Isabella Swift.

---

The wounded are still with us in hospital this Christmas season, and the County Folk Visitation Society is reminding the public that they all need special cheering and friendship, for some have been in the wards three years or more. Large numbers of soldiers are still under treatment at the 1st London General Hospital, St. Gabriel's College, Camberwell, at the 4th London, Denmark Hill, at Queen Alexandra's, Millbank, at the Special Military Orthopædic, Shepherd's Bush, at Edmonton Hospital and others. We are disposed to think that the need is less for little gifts or expensive entertainments than for the realisation of what the world will be like to these men when they are discharged, all more or less disabled, to enter the battle of life once more. If at this Christmas season all who can do so would take one disabled man as his friend, and stand by him through thick and thin, in hospital and afterwards, a load of care would be lifted from many downcast hearts.

---

The release of the meat supplies from control during the past week is likely to have a considerable influence on the institution dietary. Will there be an increase in the amount of meat used in large establishments, or will the lessons of the war, which demonstrated the extent by which the meat ration could be reduced without danger to normal workers, be acted on in days of plenty? The lazy housekeeper and the ignorant cook in combination used to act on the principle: "When in doubt play cold meat," and the result was painful to the household and disastrous to the bills. They have long been fended off this vicious expedient by restrictions on the amount of meat procurable. It will be interesting to watch whether cold meat reappears once more as only choice on the supper-table, or whether the succulent substitutes all have grown to enjoy will hold their own. Even in the old days the butcher's bill was by far the heaviest in the cost of the commissariat. At present prices it is likely to become a veritable incubus in the accounts, unless intelligent control be exercised in composing the menu.

---

The work of building and enlarging hospitals adopted in many localities is one of the most suitable forms of war memorial, but is still sadly delayed owing to the scarcity of labour and material. At Hastings it is proposed to make a start with the new hospital after Christmas, but all hope of erecting the nurses' home shown on the plans has been abandoned. Fortunately, there are two houses in close proximity to the site, which during the war were used as an auxiliary hospital. These will be adapted for the purposes of the nurses' home, and it is proposed to add considerably to the nursing staff, in order to make greatly needed alterations in duty hours. The number of beds will be raised to 100, and in anticipation of this alteration the East Sussex County Hospital has already received recognition at the hands of the College of Nursing. We hope the management will not delay shortening the hours on duty until the new hospital is ready. They call for immediate rectification, even if outside accommodation has to be obtained for the extra nurses needed to carry out reform.

